# Are Sleep Parameters and Chronotype Associated with Eating Disorder Risk? A Cross-Sectional Study of University Students in Spain

**DOI:** 10.3390/jcm13185482

**Published:** 2024-09-15

**Authors:** Tomás Olivo Martins-de-Passos, Arthur E. Mesas, Nuria Beneit, Valentina Díaz-Goñi, Fernando Peral-Martinez, Shkelzen Cekrezi, Vicente Martinez-Vizcaino, Estela Jimenez-Lopez

**Affiliations:** 1 Health and Social Research Center, Universidad de Castilla-La Mancha, 16071 Cuenca, Spain; tomas.olivo@alu.uclm.es (T.O.M.-d.-P.); valentina.diaz@uclm.es (V.D.-G.); fernando.peral@alu.uclm.es (F.P.-M.); shkelzen.cekrezi@uclm.es (S.C.); vicente.martinez@uclm.es (V.M.-V.); estela.jimenezlopez@uclm.es (E.J.-L.); 2 Facultad de Ciencias de la Salud, Universidad Autónoma de Chile, Talca 3480559, Chile; 3 Department of Psychiatry, Hospital Virgen de La Luz, 16002 Cuenca, Spain; 4 Center for Biomedical Research Network in Mental Health (CIBERSAM), Instituto de Salud Carlos III, 28029 Madrid, Spain

**Keywords:** sleep quality, night-time sleep duration, chronotype, eating disorders, university students

## Abstract

**Objectives:** Eating disorders (EDs) have emerged as a growing public health concern. However, the role of sleep in this context remains underexplored. The aim of this cross-sectional study was to determine the associations between sleep parameters and chronotype with ED risk in a sample of university students in Spain. **Methods:** ED risk was assessed via the Sick, Control, One stone, Fat, Food Questionnaire, and sleep quality was assessed via the Pittsburgh Sleep Quality Index. Other sleep parameters and chronotypes were self-reported. Sociodemographic, body composition, lifestyle, and depressive symptom data were collected. Logistic and linear regression models adjusted for the main confounders were used to estimate the odds ratios (ORs) and 95% confidence intervals (CIs) of the study associations. **Results:** A total of 403 students (70.2% female) aged 18 to 30 years participated in the study. Those reporting poor sleep quality (OR = 1.85, 95% CI 1.08–3.17, *p* = 0.025) and ≤6 h of night-time sleep duration (OR = 4.14, 95% CI 2.00–8.57, *p* < 0.01) were more likely to be at risk of EDs in the adjusted analyses. The association between night-time sleep duration and the risk of ED did not remain significant when we adjusted for sleep quality. In addition, an evening chronotype was associated with an increased risk of EDs (OR = 1.68, 95% CI 1.07–2.66, *p* = 0.039) only before adjustment for confounders. **Conclusions:** Among university students, poorer sleep quality was cross-sectionally associated with EDs. Future prospective studies are needed to examine whether promoting sleep quality may serve as an effective strategy for preventing the risk of EDs.

## 1. Introduction

Eating disorders (EDs), such as anorexia nervosa, bulimia nervosa, and binge eating disorders, are disabling, life-threatening, and costly mental conditions with significant physical and psychosocial health consequences for young adults [1,2,3]. The prevalence of probable EDs, as assessed with the Sick, Control, One, Fat, Food (SCOFF) questionnaire [4], has been estimated to be 27.6% among university students [5] and has increased since the COVID-19 pandemic [6]. Early adulthood, particularly the university years, is characterized by changes in both sleep and dietary patterns [7,8]. A high prevalence of sleep problems has been reported in this population, which may be triggered by academic pressure, irregular schedules, and lifestyle risk factors such as substance abuse, tobacco smoking, alcohol consumption, lack of physical activity, or excessive use of social media [9]. Furthermore, the prevalence of EDs increases from adolescence to young adulthood [10], highlighting the importance of investigating factors associated with the risk of these disorders in university students.

Although the specific causes of EDs are unknown, different factors have been proposed as potential predictors of their development [11,12]. The most commonly identified risk factors for EDs include sex (female), excess body weight (BMI ≥ 25 kg/m^2^), elevated concerns about body weight and self-perceived image, and compromised mental health status [13,14]. Previous studies of university students have also reported associations between EDs and lifestyle aspects such as dietary habits [15], physical fitness [16], internet addiction [17], and substance use [18].

Considering the well-established importance of sleep with respect to body composition [19], mood [20], dietary intake and food choice [21], and depression [22], previous cross-sectional studies in young adults have examined sleep as a variable associated with EDs [17,23,24,25,26,27,28,29,30,31]. Nevertheless, sleep has been examined as a secondary or adjustment variable rather than a primary variable, yielding mixed results when adjusting for covariates. To our knowledge, no study has comprehensively examined the associations of both quantitative (e.g., sleep latency, sleep efficiency, sleep disturbances, and sleep medications) and qualitative (e.g., sleep satisfaction and daytime dysfunction) components of global sleep quality [32] with the risk of EDs. This is important since these variables could be differentially associated with these conditions. Similarly, previous evidence suggested that quality may be more important for psychological and general well-being than night-time sleep duration is [33]. Thus, it seems appropriate to ascertain whether the combined associations of sleep quality and night-time sleep duration with the risk of EDs would remain independent. Furthermore, while some studies have examined the relationship between short sleep duration and the risk of EDs, it is important to also evaluate long (>8 h) night-time sleep duration, as it has been associated with adverse health outcomes [34].

Chronotype is usually defined as a person’s natural tendency regarding the time of day when they prefer to sleep or when they are most alert or energetic [35]. People with evening chronotypes tend to be more active in the evenings and prefer to sleep and wake up later, which can make it difficult to adapt to current typical work schedules [36]. It has been argued that individuals with an evening chronotype make poorer food choices, such as engaging in food addiction or binge eating in the evening [32], and could be more prone to sleep disturbances, mood and personality disorders, and mental or psychiatric problems such as EDs.

The link between sleep problems and EDs could be partially supported by both biological and psychological mechanisms. Sleep deprivation affects metabolism and immune function, both of which are critical factors in ED [37]. Poor sleep disrupts appetite-regulating hormones, increases cortisol levels, and alters brain regions involved in impulse control and reward processing, which can lead to increased cravings and disordered eating behaviors [38]. In addition, inadequate sleep has been associated with lower self-esteem and increased stress, which may contribute to restrictive eating or the use of food as a coping mechanism, thereby increasing the risk of EDs [39].

Therefore, although available evidence suggests that sleep and chronotype may be associated with the risk of EDs, there is a lack of in-depth studies investigating whether sleep quality, duration, and chronotype are related to the risk of EDs in young adults. Thus, the aims of this study were twofold: (i) to determine the associations of sleep quality, night-time sleep duration, and chronotype with the risk of EDs while controlling for potential confounding factors such as sociodemographic factors, BMI, lifestyle behaviors, and depression and (ii) to examine the combined association between sleep quality and night-time sleep duration on ED risk.

## 2. Materials and Methods

### 2.1. Study Design, Participants, and Setting

This cross-sectional study used data from the Nuts4Brain-Z (N4B-Z) study. The N4B-Z study was designed to examine the association between the consumption of nuts and depression in a sample of students from the *Universidad de Castilla-La Mancha* (UCLM) campus in Cuenca, Spain. The sample size and the calculated *post hoc* statistical power (higher than 80%) were adequate to detect significant differences in ED risk between categories of all sleep variables except for the subscores of the PSQI total sleep time and sleep disturbance, for which the statistical powers were 23.2% and 17.9%, respectively. Data collection was carried out between September and December 2023.

Using GRANMO 7.12 statistical software, a minimum sample size of 454 students was determined to detect a mean difference of 0.3 units (SD 2.04) [40] on the Center for Epidemiological Studies Depression Scale (CESD-R), with an alpha risk of 0.05 and a statistical power of 0.8. The initial population sample consisted of 463 randomly selected young adults (100.0%).

Participants were recruited from a university setting through a series of in-classroom announcements to participate in this study. The fields of study included social and legal sciences, engineering, and health sciences (nursing). The inclusion criteria were that the students were registered as regular students at the UCLM and aged between 18 and 30 years. In this study, participants with complete data for all assessed variables were included, resulting in a final sample size of 403 individuals (86.8%).

The study protocol was approved by the Research Ethics Committee of the *Hospital Virgen de la Luz*, Cuenca, Spain (report number 2023/PI1323) and complied with the Declaration of Helsinki. The students were informed of the study’s main objectives, the measurements to be taken, and the estimated time required to complete the tests. Written informed consent was acquired from each participant before the evaluation.

### 2.2. Data Collection and Variables

All participants attended scheduled visits at the MOVI Fitness Laboratory (Faculty of Nursing of Cuenca, Spain) and were assessed between 8 a.m. and 11:30 a.m., Monday to Friday.

#### 2.2.1. Risk of Eating Disorders (Dependent Variable)

We used the SCOFF questionnaire [4], a widely used screening tool for assessing suspicion of ED risk, which consists of five self-administered yes or no questions: (1) Do you make yourself vomit because you feel uncomfortably full? (2) Are you concerned that you have lost control over how much you eat? (3) Have you recently lost a stone (6 kg) in a 3-month period? (4) Do you consider yourself fat when others say you are too thin? (5) Would you say that food dominates your life? Each affirmative answer was given 1 point. A total score of two or more points, ranging from 0 to 5 points, indicates a potential risk of EDs [4]. The Spanish version of the SCOFF has been validated in primary care settings [41].

#### 2.2.2. Sleep Parameters and Chronotype (Independent Variables)

The Pittsburgh Sleep Quality Index (PSQI) [42] was used to assess the students’ overall sleep quality over the past month. It consists of 19 questions, each scored 0–3, with higher scores indicating more problems. The total score ranges from 0 to 21. The PSQI global score was used both continuously (0 to 21) and categorically (≤5: good sleep quality; >5: poor sleep quality) [42]. The Spanish version of the PSQI has good convergent and divergent validity and moderate reliability [43].

The PSQI includes seven sleep components from which the final score is derived: subjective sleep quality (1), sleep latency (2), sleep duration (3), habitual sleep efficiency (4), sleep disturbances (5), sleep medication use (6), and daytime dysfunction (7). In this study, these sleep parameters were analyzed both continuously (0- to 3-point PSQI subscores) and categorically. Components 1, 5, 6, and 7 of the PSQI were categorized as no problems (score of 0) versus any problems (scores of 1–3). Sleep latency (component 2) was categorized as ≤10, 11 to 30, or >30 min [44], and habitual sleep efficiency (component 4) was categorized using an 85% cutoff [45]. The sleep duration (component 3) categories followed the National Sleep Foundation guidelines for younger adults (7 to 9 h [h] per night) [46]. Categories were further subdivided into >7 to 8 h (baseline), ≤6 h, >6 to 7 h, and >8 h [47]. The threshold for short sleep varies across studies, ranging from 5 to 7 h, with the majority defining it as ≤6 h [48]. Night-time sleep duration was also measured continuously and was calculated as ([average bedtime—getup time]—sleep onset latency). Finally, the total sleep duration (24 h) was calculated by summing the night-time sleep duration and nap (daytime sleep) duration.

The assessment of chronotypes was conducted by asking the students whether they considered themselves to be morning or evening chronotypes. The term “morning person” was defined as an individual whose chronotype enables them to rise early and engage in activities naturally from the early morning hours. Conversely, the term “evening person” was defined as an individual who is more active in the afternoon or evening hours and performs optimally during those times of the day. The participants were asked to indicate which of the five available response options best described their chronotype: (1) clearly morning chronotype, (2) more morning than afternoon chronotype, (3) neither chronotype, (4) more afternoon than morning chronotype, and (5) clearly afternoon chronotype. The responses were classified into three categories: morning (response options 1 or 2), neutral (response option 3), and evening (response options 4 or 5) chronotypes.

#### 2.2.3. Covariates

Sociodemographic, lifestyle covariates, and depression were selected as potential confounding variables on the basis of previous evidence of their associations with both EDs [11,12,13,14,15,16,17,18] and sleep [19,20,21,22]. The sociodemographic and academic data collected included sex, age, socioeconomic status, and living situation. Responses regarding habitual residence were categorized as either living with parents or in another arrangement, which included living with a partner, in a university hall of residence, sharing a flat, or living alone. Additionally, ongoing studies were recorded, distinguishing between nursing (the only health degree on the studied campus) and other degree programs. Weight and height were objectively measured via standardized procedures. BMI was calculated (kg/m^2^) and categorized using the World Health Organization (WHO) thresholds, with BMI ≥ 25 indicating excess weight [49].

With respect to lifestyle habits, diet quality was assessed via the Mediterranean Diet Adherence Screener (MEDAS), a 14-item questionnaire scored from 0 to 14 (scores < 9 indicate poor adherence, >9 indicate good adherence) [50]. Physical activity (PA) levels were assessed via the International Physical Activity Questionnaire (IPAQ) [51]. The IPAQ categorizes physical activity into three levels. Category 1 (low) includes individuals who do not meet the criteria for the following categories and are considered inactive. Category 2 (moderate) includes individuals who meet one of the following conditions: participate in vigorous activity for at least 20 min per day for at least 3 days, participate in moderate-intensity activity or walking for at least 30 min per day for at least 5 days, or any combination of these activities for a total of at least 600 MET minutes per week. Category 3 (high) includes individuals who engage in vigorous-intensity activities for at least 3 days for a total of at least 1500 MET minutes per week, or any combination of walking, moderate, or vigorous-intensity activity for 7 or more days for a total of at least 3000 MET minutes per week [51]. The risk of problematic use of social media was assessed via the Social Network Addiction Questionnaire (ARS-6) on a 5-point scale (1 to 5), with a score of the 14th percentile or lower indicating occasional risk [52]. In addition, the Alcohol, Smoking, and Substance Involvement Screening Test (ASSIST) was used to assess the risk of substance use disorders or health consequences, with scores ≤ 10 for alcohol and ≤3 for tobacco considered low risk and higher scores (>10 for alcohol and >3 for tobacco) considered moderate to high risk [53].

Depression severity was assessed via the Beck Depression Inventory II (BDI-II), a 21-item self-reporting tool with scores ranging from 0 to 63 [54]. Depression was classified as minimal (0–13), mild (14–19), moderate (20–28), or severe (29–63) [54]. In this study, two groups were established for the analyses: the group with minimal to mild depression severity (scores of 19) and the group with moderate–severe depression (scores of 20 to 63).

### 2.3. Statistical Analysis

Descriptive data are presented as the means (M) and standard deviations (SD) for quantitative variables and frequencies (n) and percentages (%) for qualitative variables. Data screening included validity checks, evaluation for missing values, and detection of multivariate outliers through Mahalanobis distances [55]. Normality was assessed through visual and analytical methods, using normal probability plots and the Kolmogorov–Smirnov test for continuous variables. Since preliminary analyses revealed no interaction between sex × PSQI (*p* for interaction = 0.992) or field of study × PSQI (*p* for interaction = 0.987) and sleep parameters in relation to the risk of developing EDs, we analyzed these groups (i.e., female–male; nursing degree–others) together.

Student’s *t*-test (continuous variables) and the chi-square test (categorical variables) were applied to examine sample characteristics according to ED risk. To examine the associations between sleep-related indicators (independent variables) and the risk of EDs (dependent variables), crude and adjusted odds ratios (aORs) with their respective 95% confidence intervals (CIs) were calculated via logistic regression. Potential confounding variables, which are known for their associations with both sleep and EDs, were selected on the basis of the literature. Model 1 was adjusted for age, sex, and socioeconomic status. Model 2 was further adjusted for BMI and lifestyle measures (Mediterranean diet adherence, physical activity level, risk of problematic use of social media, risk of alcohol use, and risk of tobacco use), and Model 3 was also adjusted for depression. The global sleep quality score and night-time sleep duration (as continuous variables) were used as adjustment variables to explore their combined associations with the risk of EDs.

All the statistical analyses were conducted in SPSS version 28.0 (IBM Corp, Armonk, NY, USA). Significance was set at a *p*-value < 0.05.

## 3. Results

### 3.1. Description of the Sample

The characteristics of the sample are presented in Table 1 in total and by ED risk category. The mean age of the whole sample was 20.9 ± 2.4 years (70.2% women). A total of 83.9% of the sample reported a middle-to-high socioeconomic status, 74.4% did not live with their parents, and 53.6% were pursuing a non-health-related degree. Approximately one-quarter of the sample was overweight (25.8%). In terms of lifestyle factors, 73.2% of the students reported poor adherence to the Mediterranean diet, 50.4% practiced high levels of physical activity, 84.1% reported more than occasional risk of problematic social media use, 82.9% reported a low risk of alcohol use, and 62.8% reported a low risk of tobacco use. Finally, 19.1% of the students had symptoms indicating moderate-to-severe depression.

The prevalence of students at risk of EDs was 29.5%. The risk of EDs was significantly greater in females than in males (*p*-value = 0.003). Additionally, in the group of students classified as at risk of EDs (SCOFF score ≥ 2), a significantly greater proportion of individuals with excess weight, moderate–high risk of tobacco use, and moderate–severe depression symptoms was observed (*p*-value < 0.001 for all associations).

### 3.2. Descriptive Results of Sleep Parameters and Chronotype According to ED Risk

Table 2 shows the differences in sleep parameters in relation to the risk of EDs. Considering that the PSQI cutoff was >5 points, 219 (54.3%) students were classified as having poor sleep quality. Poor sleep quality was more common among students at risk of EDs (*p*-value < 0.001). The PSQI global score was significantly greater (indicating poorer sleep quality) in the ED risk group (8.15 ± 3.28) than in the group without risk of EDs (5.72 ± 2.94) (*p*-value < 0.001). With respect to the separate PSQI components, participants who reported suboptimal sleep quality (*p*-value < 0.001), >30 min of sleep onset latency (*p*-value < 0.001), shorter night-time sleep duration (≤6 h) (*p*-value < 0.001), <85% habitual sleep efficiency (*p*-value = 0.035), use of sleep medication (*p*-value < 0.001), and at least one episode of daily dysfunction in the previous month (*p*-value < 0.001) were more likely to report SCOFF scores ≥ 2.

In terms of continuous sleep parameters, those with longer sleep onset latencies (23.6 ± 21.1 vs. 32.7 ± 28.2) (*p*-value < 0.001), longer nap durations (46.2 ± 38.2 vs. 58.2 ± 51.7) (*p*-value = 0.024), and lower sleep efficiencies (88.0 ± 10.1 vs. 84.6 ± 13.5) (*p*-value = 0.007) had a greater risk of EDs. Furthermore, students who slept fewer hours were significantly more likely to be in the ED risk group (7.0 ± 1.4 versus 7.5 ± 1.1) (*p*-value < 0.001).

With respect to chronotype and sleep timing points, reporting a tendency toward an evening chronotype (*p*-value = 0.040) and reporting longer bedtimes (*p*-value = 0.030) were associated with the risk of EDs, as shown in Table 3.

### 3.3. Associations between Sleep Parameters, Chronotype, and ED Risk

Table 4 presents the unadjusted and aOR of ED risk according to each studied sleep variable. Logistic regression analysis revealed that poor sleep quality (PSQI > 5) was significantly associated with an increased risk of EDs (*p*-value < 0.001). These findings remained after we adjusted for sociodemographic factors (Model 1: aOR = 2.47, 95% CI 1.55–3.93, *p*-value < 0.001), lifestyle factors (Model 2: aOR = 2.51, 95% CI 1.52–4.17, *p*-value < 0.001), and depression (Model 3: aOR = 1.85, 95% CI 1.08–3.17, *p*-value < 0.05).

Suboptimal subjective sleep quality, more than 30 min of sleep latency, sleeping ≤6 h habitually, use of sleeping medication, and experiencing at least one episode of daytime dysfunction were significantly associated with ED risk. In contrast, total sleep time and the number of sleep disturbances were not associated with the risk of ED (*p*-value > 0.05).

Finally, the evening chronotype was significantly associated with EDs in the unadjusted model and after adjusting for sociodemographic factors (*p*-value < 0.05). However, after further adjustments for lifestyle factors (Model 2) and depression (Model 3), the association was not significant (*p*-value > 0.05). Similarly, as bedtime increased by one minute, the likelihood of ED risk increased slightly in the crude model and Model 1 (*p*-value < 0.05), losing statistical significance when controlling for BMI and lifestyle behaviors (Model 2) and depression (Model 3) (*p*-value > 0.05 in both cases).

### 3.4. Independent Associations between Sleep Quality and Night-Time Sleep Duration in Relation to the Risk of EDs

**Figure 1:**
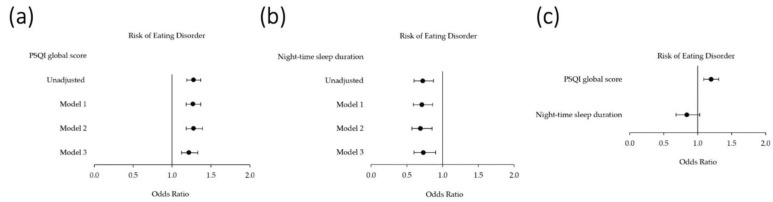
Logistic regression models of the risk of EDs (dependent variable: SCOFF score ≥ 2 points) in relation to night-time sleep duration (as a continuous variable) and the global sleep quality index (measured by the PSQI). The data are represented by dots (odds ratios) and lines (95% confidence intervals). (**a**) Crude and adjusted associations between the PSQI global score and ED risk; (**b**) crude and adjusted associations between night-time sleep duration and ED risk; (**c**) combined associations between sleep quality and night-time sleep duration in the regression model for ED groups. Note that night-time sleep duration lost statistical significance, whereas sleep quality remained significantly associated with the risk of EDs.

Figure 1 shows the independent and combined associations of global sleep quality (PSQI global score) and night-time sleep duration (continuous variable) with ED risk.

For each additional point in the PSQI score, the odds ratio of being at risk of having EDs increased by 28% (aOR = 1.28; 95% CI 1.19–1.37). Conversely, for each additional hour of night-time sleep duration, the odds ratio of being at risk of EDs decreased by 28% (aOR = 0.72; 95% CI 0.60–0.87). Both variables were independently associated with ED risk in all the models (*p*-value < 0.001 and *p*-value < 0.05, respectively). However, when the night-time sleep duration was additionally adjusted for sleep quality, its significance was lost (*p*-value > 0.05). In contrast, sleep quality remained independently associated with ED risk in all the scenarios (*p*-value < 0.001).

## 4. Discussion

To the best of our knowledge, this is the first study conducted in a population of university students addressing the relationships between parameters of sleep quality and night-time sleep duration and between chronotype and the risk of EDs. Our data suggest that poor sleep quality (PSQI > 5) and short night-time sleep duration (≤6 h) are significantly associated with the risk of EDs, regardless of potential confounders such as age, sex, socioeconomic status, BMI, lifestyle behaviors, and depression. In addition, while poor sleep quality remained associated with ED risk regardless of night-time sleep duration, night-time sleep duration was no longer associated with ED risk when sleep quality was taken into account.

In the present study, which included a population of university students, 29.5% of the participants were classified as being at risk for EDs according to the SCOFF questionnaire. These results are consistent with the most recent estimated prevalence of ED risk in college students, which is 27.6% [5]. Similarly, the proportion of students classified as having poor sleep quality in this study (54.3%) is also within the range reported for Spanish university students [56]. Furthermore, 38.5% of the participants in this study did not meet the recommended 7 h of sleep, which is also consistent with the general trend of insufficient sleep duration observed in the general population in Spain [47]. Finally, 44.2% reported an evening chronotype, which is typical of early adulthood [57].

Our results confirm previous observational studies suggesting an association between overall poor sleep quality, as measured by the PSQI, and disordered eating behaviors among university students [28,30]. In this study, there was not only a relationship between global sleep quality and the risk of EDs but also between most of the specific components analyzed. These results extend the understanding of the relationship between sleep and ED risk in university students, revealing that suboptimal subjective sleep quality, >30 min of sleep onset latency, <85% habitual sleep efficiency, at least one episode of daytime dysfunction, or the use of sleep medication, in particular, increased the likelihood of ED risk in university students.

Other studies in young adults have revealed that various sleep parameters, such as moderate insomnia [17], daytime dysfunction [24], restless sleep [25], sleep disturbances [26], fewer hours of sleep [29], and various sleep problems [58], are significantly associated with disordered eating patterns, including binge eating and poorer weight-related functioning. The available evidence offers insights into these associations. For example, global and/or ED-specific repetitive negative thoughts, such as worry and rumination, in the pre-sleep period may contribute to the development and persistence of insomnia in individuals with EDs [59]. These findings suggest a potential reciprocal relationship between prolonged sleep onset latency and the risk of EDs. Prolonged sleep onset latency may lead to shorter night-time sleep duration and lower habitual sleep efficiency, thereby worsening subjective sleep quality. Negative feelings and emotions before sleep may also increase the likelihood of using sleep medication among individuals at risk for EDs. Strong associations between substance use and ED outcomes have been reported in college settings [60]. According to self-medication theory, individuals at greater risk for EDs may use substances to cope with eating-related problems and concerns [61]. This suggests a possible cyclical pattern in which poor sleep quality exacerbates ED symptoms, potentially leading to increased use of sleep medications, which may further affect sleep. Drowsiness and fatigue are common daytime consequences of poor sleep and can result in difficulties in regulating mood and emotions [62].

Our data revealed that sleeping less than six hours increased the odds of reporting ED risk. This finding is in line with what has been reported in the literature, as individuals with short sleep durations (≤6 h) scored higher on a binge eating scale, increasing the risk of emotional eating, overeating, and weight gain, which are factors associated with EDs [58,63,64]. In studies of adolescents, each additional hour of sleep was associated with 19% lower odds of disordered eating [65], and shorter sleep duration was observed in subjects with possible EDs [66]. Among college-aged women, shorter sleep duration is one of the most important factors associated with abnormal eating attitudes [29]. However, other studies have not reported an increased risk of EDs in those who sleep less than 8 h per night [23], suggesting that the strongest association between sleep duration and the risk of EDs may be observed in students who sleep less than 6 h per night, as indicated by the results of our logistic regression analyses.

We also found no association between total sleep time (sum of night-time sleep duration and naps) and ED risk. This may be because naps could be used to compensate for night-time sleep deprivation or to cope with stressors [59]. In this sense, our results showed that those who napped more times were more likely to report ED risk. Nevertheless, the possibility that the lack of statistical significance was due to the insufficient statistical power found for the subscores of the PSQI total sleep time and sleep disturbance cannot be ruled out. Future studies with larger samples are needed to confirm these results.

Given the multifactorial onset of this disorder, it is necessary to take a broad approach to the findings discussed above, especially during young adulthood. This critical period is characterized by numerous life changes and stressors (e.g., starting college, leaving home, financial pressures, and social influences) that may influence the relationship between sleep parameters and ED risk. Nevertheless, our study revealed that both poor sleep quality and short night-time sleep duration were associated with an increased risk of EDs, even after adjustment for covariates, suggesting an independent association between sleep quality and ED risk. This was also observed in 620 Turkish university students, where poor sleep quality was significantly associated with night eating syndrome after adjustment for sociodemographic and lifestyle factors (class, residence, smoking status, and alcohol consumption) [30]. In French college students, restless sleep was significantly associated with each diagnostic category of ED according to the adjusted logistic regression model [25], and moderate insomnia heightened the probability of ED risk after adjustment for age, gender, academic year of study, curriculum, student job holder, financial difficulties, and irritable bowel syndrome [17].

Previous research in adults has also shown that poor sleep quality correlates with disordered eating behaviors after controlling for BMI [58,67], suggesting that this relationship exists across the body composition spectrum. In this context, poor sleep has been associated not only with an increased risk of EDs but also with obesity and body dissatisfaction [68,69]. Therefore, it seems reasonable to consider the possibility that the relationship between sleep and EDs may be mediated by self-perception and body satisfaction. Physiologically, sleep deprivation decreases neural responses that inhibit reward-related eating behaviors [70], leading to decreased satiety and energy expenditure. Moreover, fluctuations in sleep duration increase the consumption of high-calorie foods, heighten hunger and food cravings, and may influence susceptibility to overeating [71], potentially contributing to weight gain and obesity, which are risk factors commonly associated with EDs.

In addition, given the high degree of comorbidity between EDs and depression [72], depressed mood may also explain the association between sleep problems and ED risk. In a prospective study, persistent poor sleep quality at baseline predicted the severity of ED symptoms both directly and through the mediation of depression [73]. Previous observational research has shown that depression also mediates the relationship between insomnia and ED psychopathology [27,74]. However, these studies were conducted in clinical populations and, given that both EDs and depression are mental illnesses, the mediating role of depression in the relationship between sleep and EDs is relatively understandable. However, evidence in healthy samples remains scarce and controversial. On the one hand, depression mediates the associations between poor sleep quality, as measured by the PSQI, and disordered eating behavior among college students [30]. Nonetheless, fewer participants than in the present study reported overall poor sleep quality (36.2%), and the likelihood of EDs was assessed via the Eating Attitude Test (EAT-26), a different measure than the SCOFF. On the other hand, adult women who reported more sleep problems were significantly more likely to report lifetime binge eating even after adjusting for depression diagnosis [58]. In support of our findings, substantial evidence in the literature suggests that poor sleep quality may negatively affect mood and cognitive function, increase the difficulty of maintaining healthy eating habits, and increase the risk of emotional eating. Similarly, our study revealed that both poor sleep quality and short night-time sleep duration were correlated with confounders related to EDs, particularly depression. We hypothesize that sleep may also be indirectly associated with EDs through its association with depressive symptoms, which are strongly correlated with the SCOFF score.

In our study, an evening chronotype and delayed bedtimes were associated with increased odds of ED risk, although this difference lost statistical significance when lifestyle factors and depression were controlled for. Similar results were previously reported regarding the relationships between morning and night-time people and their risk of lifetime binge eating [58]. A review highlighted that desynchronization associated with individual chronotypes, including poorer sleep quality, shorter sleep duration, later bedtime preference, and disordered eating behaviors (e.g., late night eating, skipping breakfast, irregular mealtimes, binge eating) may contribute to the development of bulimia nervosa, binge eating disorder, and nocturnal eating disorder [75,76]. In another systematic review of observational studies evaluating young populations, evening chronotype individuals reported more late-night eating episodes, food addiction, junk food consumption, and higher BMI values than did neutral and morning chronotype individuals [77].

The mechanisms underlying the association between sleep and ED risk are complex, although some biological and psychological hypotheses have been proposed [72,78]. First, sleep and EDs are closely linked through endocrine functions and metabolic pathways. Appetite-regulating hormones such as leptin and ghrelin, which influence orexin production by the hypothalamus, are critical in maintaining the balance between sleep–wake cycles and eating behavior [38]. Elevated orexin during hunger episodes promotes wakefulness and feeding, which may lead individuals to increase food intake, increasing their susceptibility to disordered eating [79].

Sleep loss dysregulates the immune system, which may contribute to the onset or exacerbation of EDs through mechanisms such as neuroinflammation, cytokine imbalance, autoimmune processes, and disruption of the gut–brain axis [37]. Insufficient sleep may increase cortisol levels, which can increase hunger and feeding behavior while dampening the appetite-suppressing effects of leptin and increasing plasma ghrelin levels [38].

Furthermore, sleep deprivation decreases activity in higher-order cortical regions, combined with exaggerated subcortical responsiveness in the amygdala, which may lead to the selection of foods most likely to induce weight gain [80]. Sleep deprivation may affect brain areas involved in impulse control and decision-making (prefrontal cortex) [81], which could lead to increased cravings for unhealthy foods and overeating. Moreover, both sleep and eating involve the brain’s reward system, and sleep disruption can alter dopamine levels and affect reward processing [82]. These mechanisms could lead to increased cravings for unhealthy foods and overeating.

Finally, the suprachiasmatic nucleus, which regulates circadian rhythms, interacts with metabolic centers in the brain that control hunger, satiety, and energy balance (such as the hypothalamus and brainstem) as well as hedonic centers involved in the brain’s reward system (nucleus accumbens and ventral tegmental area) [83]. Moreover, desynchronization between the internal circadian clock and environmental light–dark cycles leads to dysregulation of melatonin release [84], resulting in sleep disturbances. Melatonin, which interacts with serotonin, a neurotransmitter that regulates mood, appetite, and sleep, also plays a critical role in mood regulation. This misalignment of biorhythms affects the timing of hunger and satiety signals, leading to irregular eating patterns, such as late-night meals or prolonged fasting periods. These disruptions can increase the likelihood of overeating or binge eating [76,85].

From a psychological perspective, poor sleep quality could contribute to the risk of EDs because of its association with lower self-esteem and reduced confidence in one’s body and abilities [39]. This negative self-perception may lead individuals to restrict food intake in an effort to improve their appearance [86]. Stress resulting from poor sleep may perpetuate the use of food as a coping mechanism for emotional distress. This may manifest in two possible ways: first, through episodes of overeating, which may be used to self-medicate or combat fatigue and low energy, and second, through episodes of food restriction, which may be used as an attempt to regain a sense of control [87].

To our knowledge, no cross-sectional studies have examined the association between night-time sleep duration and ED risk independently of sleep quality. In contrast to our hypothesis, night-time sleep duration was not independently associated with ED risk after controlling for sleep quality.

Although our study revealed that each additional hour of night-time sleep significantly decreased the odds ratio for ED risk, current evidence suggests that both excessively short and long sleep durations may be detrimental. A recent umbrella review and meta-analysis of prospective studies revealed a significant association between long sleep duration and increased risk of all-cause mortality [34]. Sleep quality may be a more important indicator of mental health than sleep quantity is [88]. For example, among 25,962 participants in a cross-sectional study, the risk of depression significantly increased when the duration of sleep was ≥8 h [89]. Similarly, sleep quality appears to be a more critical risk factor for mental health among college students. An observational study comparing sleep quality, sleep duration, and chronotype as predictors of self-reported mental health identified sleep quality as one of the strongest independent predictors [90]. This finding is consistent with our results because poor sleep quality significantly predicts ED risk regardless of sleep duration. The reasons for these findings are complex. Notably, sleep quality encompasses several sleep parameters, including sleep duration. In our study, all of these components (except PSQI component 4, sleep disturbances) were significantly associated with the risk of EDs. As noted by other authors, self-reported sleep duration may be subject to various biases [91]. These findings suggest that extreme night-time sleep duration may indicate poor sleep quality. Such extremes are likely to reflect poor sleep quality rather than truly short or long durations, partly explaining the U-shaped associations between sleep duration and health outcomes [92]. Nevertheless, some studies have shown that the effects of sleep duration and sleep quality on health outcomes are not simply additive [93,94], supporting the need to consider both aspects of sleep whenever possible.

These findings underscore the possible role of improving sleep quality as a primary prevention strategy for EDs. This can be of importance from a public health perspective, given the high potential for change in sleep quality throughout lifestyle interventions, making this a plausible approach at the population level. In this context, the National Sleep Foundation from the United States of America has recommended several sleep hygiene practices, which have proven effective at both improving sleep quality and reducing the occurrence of psychological symptoms associated with EDs [95].

Several limitations must be considered when our results are interpreted. First, most participants were female (70.2%), who are more prone to sleep problems and ED risk [5,96]. In addition, the sample included a substantial percentage of nursing students (46.4%), which may affect the representativeness of the sample and limit the generalizability of the results to the broader student population, since the results from other studies have highlighted a high prevalence of ED symptomatology among students enrolled in health-related degrees [97,98]. Future research should aim to include more male participants and students from non-health-related fields to reduce potential selection bias. Second, the study relied on self-reported tools to assess sleep quality and chronotype, which may introduce bias due to the subjective nature of the information. Future research would benefit from incorporating more objective measures, such as actigraphy [99]. Third, this was a cross-sectional study, making it impossible to establish a causal relationship between sleep quality and ED risk. Evidence suggests that sleep and ED may be bidirectionally related [59,100,101] and it remains unclear whether poor sleep quality leads to ED risk or vice versa. Future studies with a prospective design are necessary to better understand the direction of these relationships.

## 5. Conclusions

In conclusion, this study examined the associations of sleep quality and duration and chronotype with ED risk in a sample of university students. The results indicate that poor sleep quality and short night-time sleep duration (≤6 h) are associated with increased odds of reporting ED risk, independent of potential confounders such as sociodemographic factors, BMI, lifestyle habits, and depressive symptoms. In light of these findings, it is necessary to emphasize the importance of overall good sleep quality (i.e., sleep onset, maintenance, rest, no need for additional sleep, sufficient objective sleep depth) rather than simply prolonging night-time sleep duration to reduce the likelihood of the risk of EDs.

These findings have important public health implications and underscore the importance of maintaining good sleep health to reduce the risk of EDs. The increasing prevalence of ED among university students calls for urgent planning of preventive interventions and support for those in need. Early detection and treatment significantly reduce symptoms and improve the chances of recovery. Routine screening for EDs in asymptomatic populations could facilitate early detection and treatment, thereby reducing future morbidity and mortality. Therefore, assessing poor sleep in ED prevention protocols may reduce the development of EDs and improve treatment efficacy, as persistent sleep problems increase comorbidity and attrition from standard interventions. Overall, prospective studies are needed to evaluate whether incorporating sleep management into standard ED preventive measures improves symptoms, treatment outcomes, and overall quality of life.

## Figures and Tables

**Table 1:** Characteristics of the sample according to the risk of eating disorders.

	Total, n (%)	No Risk of EDs *, n (%)	At Risk of EDs *, n (%)	*p*-Value
Total	403 (100.0)	284 (70.5)	119 (29.5)	
Sex				**0.003**
Female	283 (70.2)	187 (66.1)	96 (33.9)	
Male	120 (29.8)	97 (80.8)	23 (19.2)	
Age (years)				0.886
18 to 21	273 (67.7)	193 (70.7)	80 (29.3)	
21–30	130 (32.3)	91 (70.0)	39 (30.0)	
Socioeconomic status				0.405
Low	65 (16.1)	43 (66.2)	22 (33.8)	
Medium–High	338 (83.9)	241 (71.3)	97 (28.7)	
Living situation				0.108
Living with both parents	103 (25.6)	79 (76.7)	24 (23.3)	
Living in another situation	300 (74.4)	205 (68.3)	95 (31.7)	
University studies				0.864
Nursing degree	187 (46.4)	131 (70.1)	56 (29.9)	
Other non-health degrees	216 (53.6)	153 (70.8)	63 (29.2)	
BMI status (kg/m^2^)				**<0.001**
No excess weight (BMI < 25)	299 (74.2)	230 (76.9)	69 (23.1)	
Excess weight (BMI ≥ 25)	104 (25.8)	54 (51.9)	50 (48.1)	
Mediterranean diet adherence				0.052
Poor adherence	295 (73.2)	200 (67.8)	95 (32.2)	
Good adherence	108 (26.8)	84 (77.8)	24 (22.2)	
Physical activity level				0.503
Low	59 (14.6)	39 (66.1)	20 (33.9)	
Moderate	141 (35.0)	104 (73.8)	37 (26.2)	
High	203 (50.4)	141 (69.5)	62 (30.5)	
Risk of problematic use of social media				0.244
Occasional risk	64 1(5.9)	49 (76.6)	15 (23.4)	
More than occasional	339 (84.1)	235 (69.3)	104 (30.7)	
Risk of alcohol use				0.103
Low risk	334 (82.9)	241 (72.2)	93 (27.8)	
Moderate–high risk	69 (17.1)	43 (62.3)	26 (37.7)	
Risk of tobacco use				**<0.001**
Low risk	253 (62.8)	193 (76.3)	60 (23.7)	
Moderate–high risk	150 (37.2)	91 (60.7)	59 (39.3)	
Depression				**<0.001**
Minimal–mild (0 to 19)	326 (80.9)	253 (77.6)	73 (22.4)	
Moderate–severe (20 to 63)	77 (19.1)	31 (40.3)	46 (59.7)	

The data are expressed as numbers (percentages). BMI, body mass index; EDs, eating disorders. Bold values indicate a *p*-value < 0.05. * Risk of eating disorders is measured with the SCOFF score, considering a score < 2 as no risk of EDs and ≥2 as risk of EDs [4].

**Table 2:** Sleep parameters in total and according to the risk of eating disorders (SCOFF score ≥ 2).

Sleep Parameters	Total	No Risk of EDs *	At Risk of EDs *	*p*-Value
PSQI global score (categories)				**<0.001**
≤5 (good sleep quality)	184 (45.7)	149 (81.0)	35 (19.0)	
>5 (poor sleep quality)	219 (54.3)	135 (61.6)	84 (38.4)	
PSQI global score (continuous), M ± SD	6.44 ± 3.24	5.72 ± 2.94	8.15 ± 3.28	**<0.001**
Subjective sleep quality, n (%)				**<0.001**
Optimal	284 (70.5)	228 (80.3)	56 (19.7)	
Suboptimal	119 (29.5)	56 (47.1)	63 (52.9)	
PSQI subjective sleep quality subscore, M ± SD	1.18 ± 0.70	1.04 ± 0.67	1.51 ± 0.65	**<0.001**
Sleep onset latency (min), n (%)				**<0.001**
≤10	126 (31.3)	99 (78.6)	27 (21.4)	
11 to 30	189 (46.9)	138 (73.0)	51 (27.0)	
>30	88 (21.8)	47 (53.4)	41 (46.6)	
Continuous (min), M ± SD	26.3 ± 23.8	23.6 ± 21.1	32.7 ± 28.2	**0.002**
PSQI sleep latency subscore, M ± SD	1.37 ± 0.93	1.24 ± 0.89	1.66 ± 0.94	**<0.001**
Night-time sleep duration (hours), n (%)				**<0.001**
≤6	60 (14.9)	27 (45.0)	33 (55.0)	
>6 to 7	95 (23.6)	67 (70.5)	28 (29.5)	
>7 to 8	148 (36.7)	116 (78.4)	32 (21.6)	
>8	100 (24.8)	74 (74.0)	26 (26.0)	
Continuous (hours), M ± SD	7.3 ± 1.2	7.5 ± 1.1	7.0 ± 1.4	**<0.001**
PSQI sleep duration subscore, M ± SD	1.08 ± 0.87	0.98 ± 0.82	1.33 ± 0.93	**<0.001**
Nap duration (min), M ± SD	49.8 ± 43.0	46.2 ± 38.2	58.2 ± 51.7	**0.024**
Total sleep time (hours), M ± SD	8.1 ± 1.4	8.2 ± 1.2	8.0 ± 1.6	0.134
Sleep efficiency (percentage), n (%)				**0.035**
High efficiency (≥85)	269 (66.7)	198 (73.6)	70 (26.1)	
Low efficiency (<85)	134 (33.3)	86 (64.2)	49 (36.3)	
Continuous (percentage), M ± SD	87.0 ± 11.3	88.0 ± 10.1	84.6 ± 13.5	**0.007**
PSQI sleep efficiency subscore, M ± SD	0.50 ± 0.84	0.42 ± 0.75	0.70 ± 1.00	**0.007**
Sleep disturbances, n (%)				0.302
No disturbance	111 (27.5)	74 (66.7)	37 (33.3)	
Disturbance at least once	292 (72.5)	210 (71.9)	82 (28.1)	
PSQI sleep disturbances subscore, M ± SD	0.89 ± 0.65	0.87 ± 0.62	0.92 ± 0.73	0.577
Use of sleeping medication, n (%)				**<0.001**
Never	332 (82.4)	246 (74.1)	86 (25.9)	
Sometime	71 (17.6)	38 (53.5)	33 (46.5)	
PSQI sleep medication subscore, M ± SD	0.32 ± 0.78	0.24 ± 0.68	0.52 ± 0.94	**0.003**
Daytime dysfunction, n (%)				**<0.001**
Never	100 (24.8)	89 (89.0)	11 (11.0)	
At least one episode	303 (75.2)	195(64.4)	108 (35.6)	
PSQI daytime dysfunction subscore, M ± SD	1.10 ± 0.82	0.93 ± 0.78	1.51 ± 0.79	**<0.001**

Data are expressed as the number (n) and percentage (%) of individuals, except when the indicated mean ± standard deviation (M ± SD) is used. EDs, eating disorders; PSQI, Pittsburgh Sleep Quality Index. Bold values indicate a *p*-value < 0.05. * Risk of eating disorders is measured with the SCOFF score, considering a score < 2 as no risk of EDs and ≥2 as risk of EDs [4].

**Table 3:** Chronotype and sleep timing points in total and according to the risk of eating disorders (SCOFF score ≥ 2).

	Total	No Risk of EDs	At Risk of EDs	*p*-Value
Chronotype, n (%)				**0.040**
Neutral chronotype	45 (11.2)	35 (77.8)	10 (22.2)	
Morning chronotype	180 (44.7)	135 (75.0)	45 (25.0)	
Evening chronotype	178 (44.2)	114 (64.0)	64 (36.0)	
Bedtime (hh:mm ± min), M ± SD	00:27 ± 01:05	00:22 ± 01:00	00:39 ± 01:14	**0.030**
Get-up time (hh:mm ± min), M ± SD	08:12 ± 01:04	08:13 ± 0:58	08:10 ± 01:15	0.709

Data are expressed as the number (n) and percentage (%) of individuals, except when the indicated mean ± standard deviation (M ± SD) is used. EDs, eating disorders. Bold values indicate a *p*-value < 0.05.

**Table 4:** Logistic regression models with sleep parameters (independent variables) and the risk of eating disorders (dependent variable) in university students.

Sleep Parameters	Unadjusted Model	Model 1	Model 2	Model 3
	Crude OR (95% CI)	aOR (95% CI)	aOR (95% CI)	aOR (95% CI)
PSQI score (categories)				
≤5 (good sleep quality)	1.00	1.00	1.00	1.00
>5 (poor sleep quality)	2.65 (1.68–4.19) **	2.47 (1.55–3.93) **	2.51 (1.52–4.17) **	1.85 (1.08–3.17) *
Subjective sleep quality				
Optimal	1.00	1.00	1.00	1.00
Suboptimal	4.58 (2.88–7.28) **	4.41 (2.76–7.05) *	4.50 (2.69–7.52) **	3.42 (1.99–5.88) **
Sleep onset latency (min)				
≤10	1.00	1.00	1.00	1.00
11 to 30	1.36 (0.80–2.31)	1.34 (0.78–2.29)	1.26 (0.71–2.23)	1.18 (0.65–2.16)
>30	3.20 (1.76–5.81) **	3.01 (1.65–5.51) **	3.24 (1.69–6.20) **	2.84 (1.45–5.55) *
Night-time sleep duration (hours)				
≤6	4.43 (2.33–8.42) **	4.33 (2.20–8.30) **	5.13 (2.52–10.42) **	4.14 (2.00–8.57) **
>6 to 7	1.52 (0.84–2.73)	1.46 (0.80–2.65)	1.53 (0.80–2.91)	1.38 (0.71–2.71)
>7 to 8	1.00	1.00	1.00	1.00
>8	1.27 (0.70–2.30)	1.20 (0.65–2.18)	1.23 (0.65–2.34)	1.24 (0.64–2.41)
Total sleep time (hours, range: 3.5–12)	0.87 (0.74–1.02)	0.85 (0.73–1.00)	0.80 (0.68–0.95)	0.85 (0.71–1.01)
Sleep efficiency (%)				
High efficiency (≥85)	1.00	1.00	1.00	1.00
Low efficiency (<85)	1.61 (1.03–2.51) *	1.53 (0.98–2.41)	1.65 (1.01–2.69) *	1.33 (0.79–2.23)
Number of sleep disturbances				
None	1.00	1.00	1.00	1.00
At least once	0.78 (0.49–1.25)	0.79 (0.49–1.27)	0.76 (0.46–1.26)	0.72 (0.42–1.22)
Use of sleeping medication				
Never	1.00	1.00	1.00	1.00
Sometime	2.48 (1.47–4.21) **	2.62 (1.53 4.50) **	2.82 (1.58–5.02) **	2.43 (1.32–4.45) *
Daytime dysfunction				
Never	1.00	1.00	1.00	1.00
At least one episode	4.48 (2.30–8.75) **	4.13 (2.10- 8.11) **	4.35 (2.13–8.88) **	3.12 (1.50–6.49) *
Chronotype				
Neutral chronotype	1.00	1.00	1.00	1.00
Morning chronotype	0.86 (0.39–1.87)	0.81 (0.37–1.79)	0.76 (0.33–1.74)	0.65 (0.27–1.58)
Evening chronotype	1.68 (1.07–2.66) *	1.63 (1.02–2.58) *	1.51 (0.92–2.48)	1.46 (0.87–2.45)
Bedtime (hh:mm, range: 08:30 p.m., 05:00 a.m.)	1.00 (1.00–1.01) *	1.00 (1.00–1.01) *	1.00 (1.00–1.01)	1.00 (0.99–1.01)
Get-up time (hh:mm, range: 04:00 a.m., 02:00 p.m.)	1.00 (0.99–1.00)	1.00 (0.99–1.00)	1.00 (0.99–1.00)	1.00 (0.99–1.00)

Model 1: adjusted for sex, age, and socioeconomic status. Model 2: Adjusted Model 1 plus adjustment for body mass index (BMI), Mediterranean diet adherence, physical activity level, risk of problematic use of social media, risk of alcohol use, and risk of tobacco use. Model 3: Adjusted Model 2 plus depression. A cutoff point for eating disorders ≥2 points on the Sick, Control, One, Fat, and Food (SCOFF) questionnaire. aOR, adjusted odds ratio; CI, confidence interval; OR, odds ratio. * *p*-value < 0.05. ** *p*-value < 0.001. Interaction test: Sex × sleep quality index (PSQI), *p* for interaction = 0.992. Field of study × PSQI, *p* for interaction = 0.987.

## Data Availability

Data will be available upon reasonable request to the corresponding author.
